# Inferring Local Protein Structural Similarity from Sequence Alone

**DOI:** 10.1021/acs.jcim.6c00114

**Published:** 2026-05-02

**Authors:** Zinnia Ma, Javier Espinoza Herrera, Elsy Buitrago-Delgado, Neville P. Bethel, Adrian Jinich

**Affiliations:** † 8784 Department of Bioengineering, University of California, San Diego 92093, California, United States; ‡ Department of Biochemistry and Molecular Biophysics, University of California, San Diego 92093, California, United States; § Skaggs School of Pharmacy and Pharmaceutical Sciences, University of California, San Diego 92093, California, United States; ∥ School of Medicine Division of Regenerative Medicine, University of California, San Diego 92093, California, United States

## Abstract

Detecting structural similarity at the local level between proteins is central to understanding function and evolution, yet most approaches require 3D models. In this work, we show that protein language models (pLMs), solely using sequence data as input, implicitly capture fine-grained structural signals that can be leveraged to identify such similarities. By mean-pooling residue embeddings over sliding windows and comparing them across proteins with cosine similarity, we find diagonal patterns that reflect locally aligned regions, even without sequence identity. Building on this insight, we introduce a framework for detecting locally aligned structural regions directly from sequences, supporting the development of scalable methods for structural annotation and comparison. The effectiveness and scalability of this framework are further demonstrated through a case study identifying SRC homology 3 (SH3) domains within a large-scale PDB subset, where our approach successfully recovered structurally conserved motifs across diverse sequence contexts. Ultimately, this work provides a lightweight alternative to structure-based methods, paving the way for high-throughput structural discovery using sequence data alone.

## Graphical abstract

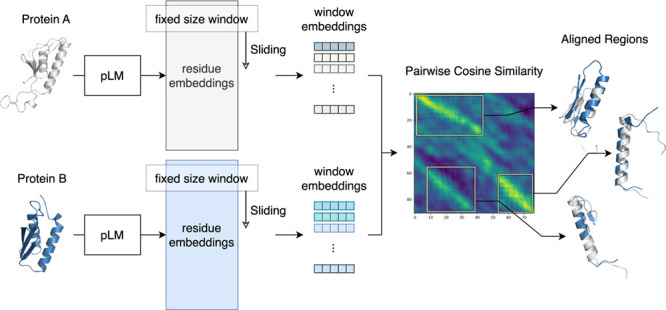

## Introduction

Understanding protein function and evolution often hinges on structural similarity, as proteins with comparable folds often perform related biochemical activities, even in the absence of sequence similarity.^1^ Detecting such similarities is also critical in practice, supporting annotation of uncharacterized proteins, rational engineering, and drug discovery.^2–5^ Traditional methods for identifying structural similarity, such as TM-align and DALI (Distance Matrix Alignment), align three-dimensional protein models obtained from experimental determination or computational predictions.^6–8^ More recent tools, such as Foldseek, generate compact representations of protein structures to enable rapid comparisons; however, they still require a three-dimensional structural model as input.^9^

In contrast, methods that solely rely on sequence have emerged; however, these are generally limited to detecting global fold-level similarities and remain insufficient for capturing local structural relationships.^10–12^ More recently, pLMs such as ESM and ProtTrans have overcome this limitation by encoding structural and functional properties directly from sequence.^13–15^ Despite being trained solely on large protein sequence corpora, their embeddings have been successfully applied to diverse downstream tasks, including secondary structure prediction, remote homology detection, contact prediction, and protein function annotation.^16–19^ Thus, the ability to recover local structural similarity directly from a sequence offers a practical advantage that bypasses the need for structural models. This motivates the central question of this work: whether pLM embeddings contain sufficient information to identify the local structural similarity between proteins.

To address this question, we developed a sequence-only framework that detects locally similar structural regions by using pLM-derived embeddings. Our approach computes sliding window embeddings for two proteins, constructs a window-similarity matrix, enhances the resulting signal using a sigmoid-based transformation, and then identifies high-scoring local regions using a Smith–Waterman-style alignment procedure. This enables us to recover segments that are similar in structure, even when the full-length sequences differ substantially. We tested a series of pLMs, including ProtT5, ESM-2 (3B) and (15B), ESM-3 (7B), CARP, and ProstT5.^12,13,15,20,21^ Among them, ProtT5 and ProtT5 provided the clearest local structural signals. Applied to the MALISAM benchmark of structural analogs (protein pairs that share similar local folds despite lacking common ancestry),^22^ our approach identifies local regions with high structural similarity, achieving TM-scores above 0.5 in many cases despite low global structural similarity. Comparative experiments further show that our method outperforms baseline methods based on residue alignment and predicted 3Di sequences, demonstrating that local structural information is well encoded in sequence-only ProstT5 embeddings.

## Related Work

Foldseek represents the most efficient and widely used method for large-scale structural alignment. This method encodes protein structures as 3Di (3D interaction) sequences, which represent each residue by a structural state letter based on the 3D structure, facilitating high-throughput and accurate comparisons.^9^ Recent sequence-based approaches such as ProstT5 and ESM-2 3B 3Di aim to bypass the need for structural data by directly translating amino acid sequences into predicted 3Di representations, thereby leveraging Foldseek’s powerful alignment algorithm.^11,12^ However, due to the limited prediction accuracy of 3Di tokenization, currently around 60%, these methods are better suited for global fold-level retrieval tasks than fine-grained fragment-level structural searches.

DeepBLAST is another existing model that could structurally align proteins using only sequence information.^23^ This method is capable of aligning structurally homologous domains with low sequence identity such as duplicated Annexin domains. However, because it is based on the Needleman–Wunsch algorithm, which is inherently designed for global alignment, DeepBLAST faces intrinsic limitations when homologous regions are restricted to small local segments. In such cases, enforcing a global alignment may dilute the signal of local structural similarity, making it harder to detect.

To address the challenge of capturing these nuanced local structural signals, we draw inspiration from the concept of neighborhood processing through sliding windows.^24^ As a robust mechanism for aggregating local context and refining signal extraction, the sliding window paradigm has demonstrated success in protein sequence analysis. For instance, SWING^25^ utilizes a sliding window to traverse potential interacting subsequence pairs, thereby accounting for inherent context specificity and enhancing model generalization. Building upon this intuition, our work validates that the sliding window approach is exceptionally effective at capturing local protein structural information, providing the granularity that global alignment and discrete tokenization often overlook.

## Methods

### Capturing the Alignment Signal via Pairwise Cosine Similarity of Sliding Window Embeddings

Given a protein sequence of length *n*, we first obtain per-residue embeddings of shape *n* × *d* using a pLM. To extract local contextual representations, we apply a sliding window of size *w* across the sequence, yielding in *n* – *w* + 1 overlapping segments. For each window, we perform mean pooling over its corresponding *w* × *d* local residue embeddings to produce a single *d*-dim window-level embedding. For a pair of proteins, we then compute the cosine similarity between all pairs of window embeddings, generating a matrix in which each entry reflects the similarity between specific local regions. This matrix serves as the basis for the downstream analysis of structurally analogous regions.

### Enhancing Alignment Signal with Sigmoid-Based Transformation

To convert the cosine similarity matrix into a reward matrix with enhanced contrast between aligned and misaligned regions, we applied a nonlinear transformation function to emphasize high-similarity regions while penalizing low-similarity ones. Specifically, we used a scaled sigmoid function:

$$R(x)=\mathrm{scale}\cdot \left(\frac{1}{1+e^{-\mathrm{sharpness}(x-\mathrm{midpoint})}}-0.5\right)\cdot 2$$

where *x* ∈ [−1, 1] is the normalized cosine similarity. Before the transformation was applied, the raw similarity matrix was linearly rescaled to this range. The parameters were chosen to balance both signal sensitivity and robustness to noise.

### Detecting the Alignment Regions Based on the Smith–Waterman Algorithm

The Smith–Waterman algorithm is a dynamic programming method for local sequence alignment. In our approach, we adapt a Smith–Waterman-style algorithm to identify structurally aligned regions between a pair of proteins.^26^ Specifically, we use the reward matrix obtained by applying the transformation described above to the raw cosine similarity matrix as the scoring basis in the Smith–Waterman framework (Figure 1). In this way, window pairs with high cosine similarity receive high match rewards, while dissimilar pairs are assigned strong mismatch penalties. To balance insertions and deletions, we introduced an additional indel penalty term. By completing the scoring matrix and performing traceback, we extracted the highest-scoring aligned windows and mapped them back to the corresponding protein regions.

**Figure 1:**
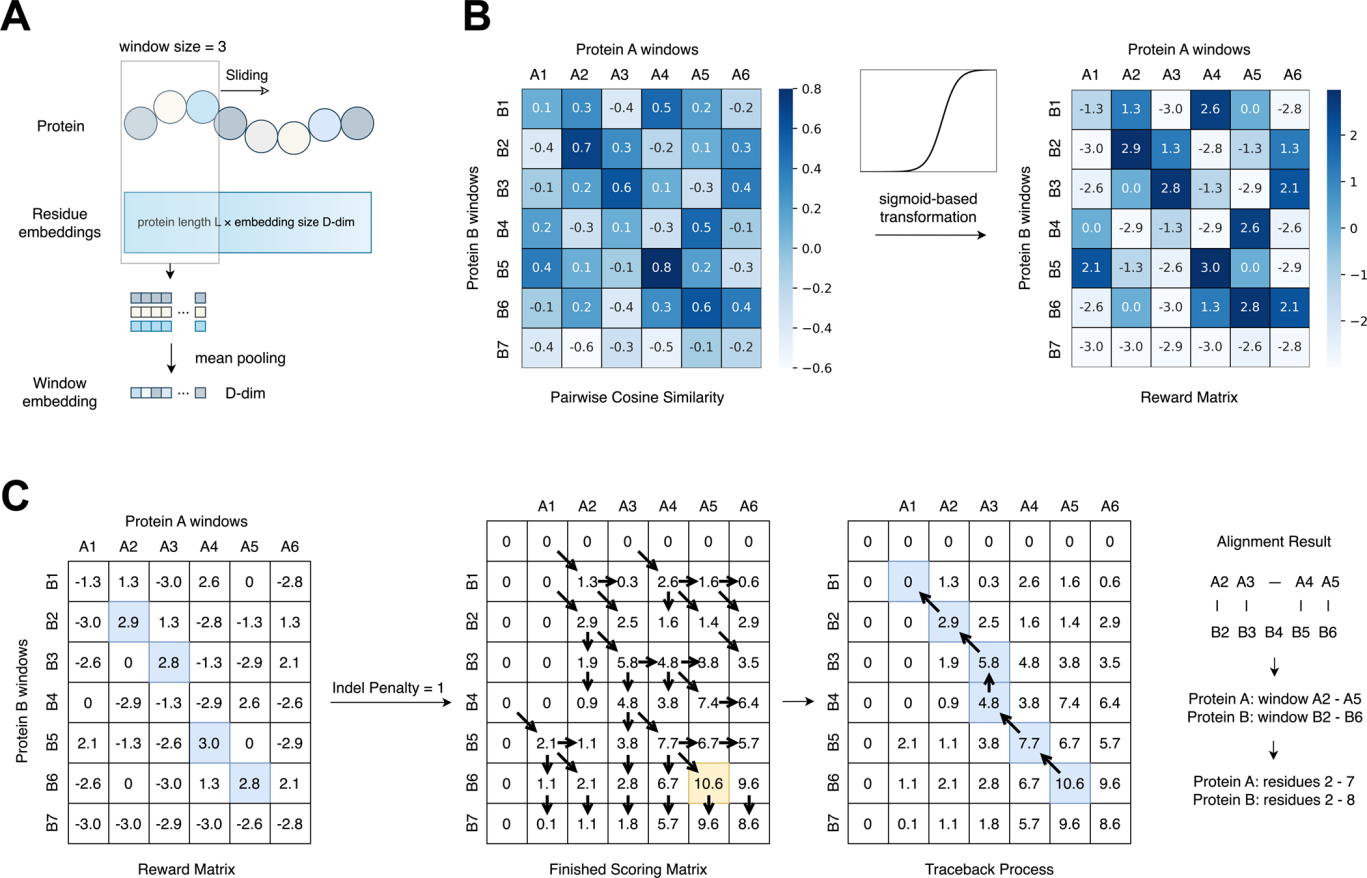
Workflow of the alignment method. (A) Extract window embeddings from pLM-derived residue embeddings. (B) Sigmoid-based transformation for signal enhancement. (C) Alignment based on the Smith–Waterman algorithm using a predefined reward matrix. Cosine similarities between sliding window embeddings are transformed into a reward matrix, which is then used by a Smith–Waterman-style algorithm to identify structurally aligned regions. The figure demonstrates how our method reveals alignment signals embedded in the original pairwise cosine similarity matrix. The traceback path and corresponding alignment signal are highlighted in blue, while the maximum score in the scoring matrix is marked in yellow. The pseudocode of the algorithm is provided in Supplementary Methods, Algorithm 1. It should be noted that the parameters displayed in this schematic (e.g., indel penalty = 1) are selected for illustrative clarity and may differ from the values used in the actual experiments (e.g., indel penalty = 10).

### Hyperparameter Selection and Evaluation Metrics

To determine the optimal configuration for our workflow, we conducted a systematic grid search over the hyperparameters of the sigmoid-based transformation. The midpoint was varied from 0 to 1 with a step size of 0.1, while both sharpness and scale were varied from 1 to 10 with a step size of 1, resulting in a total of 1100 combinations. For each combination, we applied the proposed workflow to every protein pair in the data set to identify a candidate region pair. The performance was then evaluated based on the length of the detected regions and their structural similarity. Structural similarity was measured using the TM-score, where scores >0.5 indicate a shared fold and scores <0.2 represent random similarity. This comprehensive evaluation allowed us to identify the configuration that best balances alignment accuracy with biologically meaningful region lengths.

## Experiments

We evaluated our approach with the MALISAM database, a curated benchmark of 130 protein pairs from experimentally determined structures.^22^ Unlike homologous proteins, which share features through common ancestry, MALISAM emphasizes analogous proteins that evolved similar folds independently. Due to their lack of shared ancestry, these protein pairs exhibit significantly lower sequence identity compared to structural homologues. Each pair consists of two sequence regions that align structurally even though their corresponding PDB structures differ at the global fold level. Each aligned region was defined using a combination of manual inspection and computational annotation. To curate the data set, hybrid and core motifs were first identified across SCOP domains and then used as queries in DALI searches against a culled PDB set restricted to <50% sequence identity, ensuring that the resulting analog pairs reflect structural rather than evolutionary similarity. These motifs provide a rigorous standard for testing whether sequence-based embeddings can recover structural similarity.

### Heatmaps of Embedding Cosine Similarity Reveal Structurally Analogous Regions

To test whether pLM embeddings capture structural similarity signals in the absence of high sequence identity, we generated pairwise cosine similarity matrices by comparing sliding windows across two analogous protein sequences. Each window corresponds to the average per-residue embedding from ProtT5 over five amino acids. In these matrices, higher cosine similarity values appear as continuous diagonals, suggesting alignment-like relationships between protein regions. Notably, these patterns emerge even when the sequences lack detectable homology, as with protein pairs in the MALISAM data set containing structural analogs.

The two representative examples shown in Figure 2 illustrate these observations. For the pair 1a2z-1ghh in panel A, the diagonals in the similarity matrix align with α-helices in the 3D structures, showing that embedding-derived similarities directly coincide with secondary structure features. In contrast, the pair 1slc–1sq9 in panel B highlights how the predominance of β-sheets gives rise to multiple diagonals, again capturing structural alignment despite sequence divergence. Together, these cases demonstrate that windowed averages of protein embeddings effectively reflect three-dimensional organization, reinforcing their value in identifying structurally analogous regions in proteins with little to no sequence similarity.

**Figure 2:**
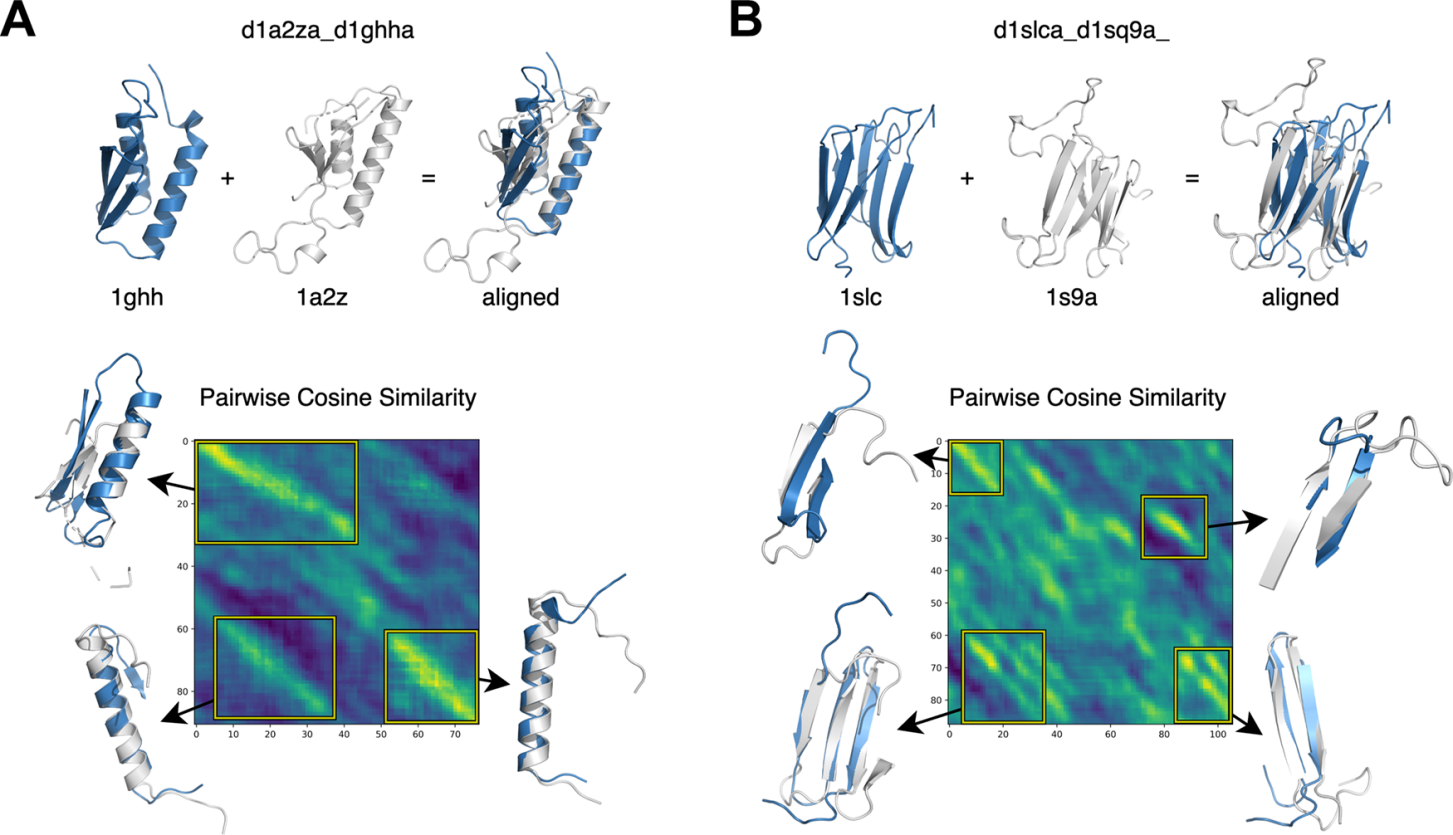
Heatmaps of cosine similarity across protein sequence pairs calculated using a sliding window of 5 residues. The *y*-axis represents the starting position of each window in the first protein, while the *x*-axis represents the starting position in the second protein. The heatmaps highlight structural correspondence, exemplified by (A) α-helices and (B) β-sheets in different protein pairs.

### Comparing the Performance of Different pLMs in Capturing Fine-Grained Structural Information

We continued by comparing the performances of different pLMs in capturing structural similarity signals. For each model, we compute pairwise cosine similarity matrices for all aligned protein pairs in the MALISAM data set using a sliding window approach with a window size of 5. The resulting matrices are visualized as heatmaps. Representative examples are shown in Figures 3 and S1. This qualitative comparison enables us to visually assess the strength and clarity of the structural signals captured by different pLMs. To more reliably assess the correspondence between the captured signals and the ground truth alignments, we performed a quantitative evaluation. Specifically, we compute precision, recall, and F1 score by comparing the observed signal patterns in the heatmaps to the ground truth structural alignments. The detailed results are presented in Figure S2.

**Figure 3:**
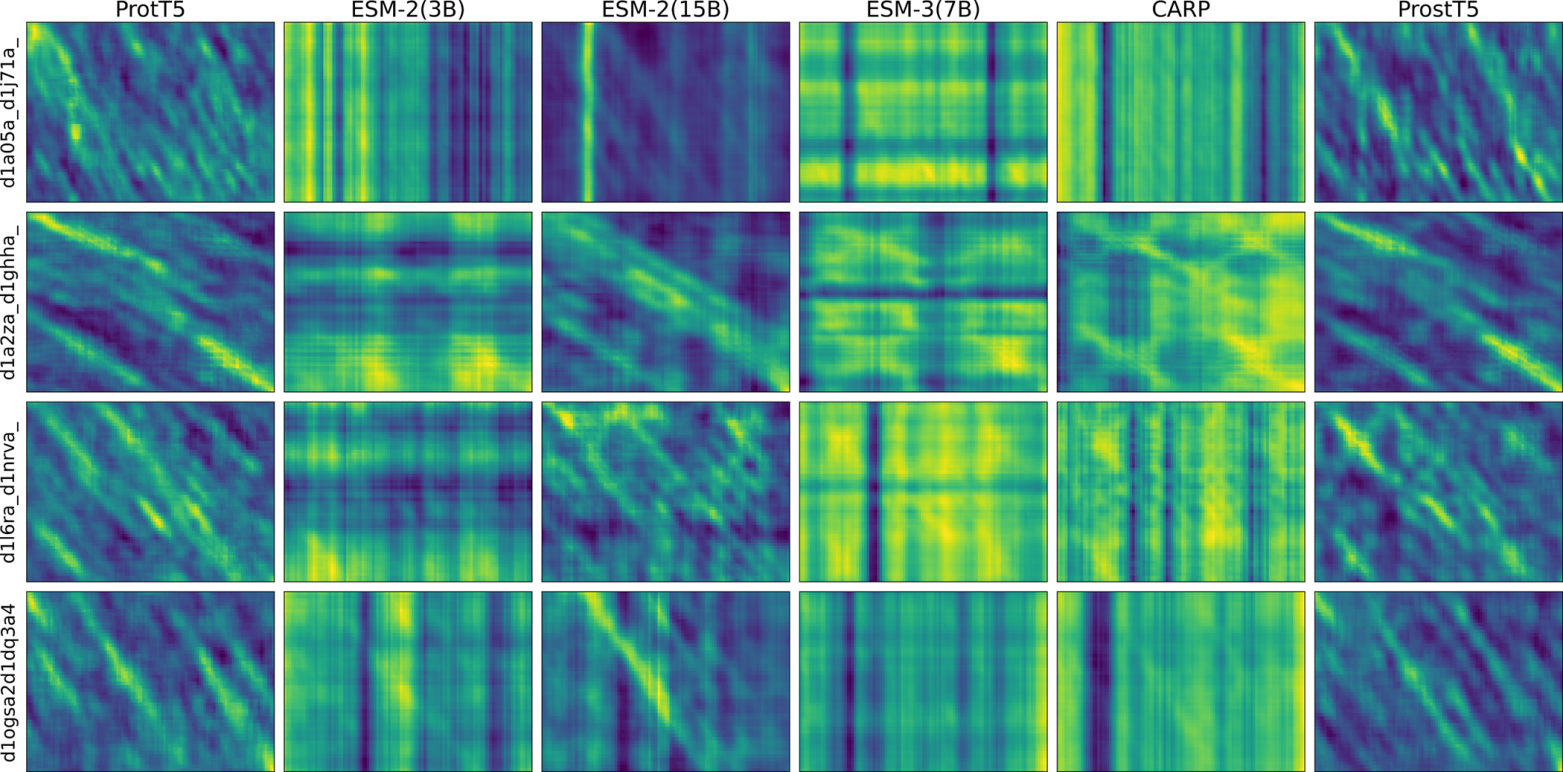
Comparison of pairwise cosine similarity patterns across different pLMs. Each column corresponds to a pLM: ProtT5, ESM-2 (3B), ESM-2 (15B), ESM-3 (7B), CARP, and ProstT5, from left to right. Each row represents a randomly selected aligned protein pair from the MALISAM data set, with the pair name shown on the left. The matrices are computed using a sliding window of size 5 and visualized to assess the reliability and clarity of alignment signals produced by different models.

The pLMs evaluated are ProtT5, ESM-2 (3B), ESM-2 (15B), ESM-3 (7B), CARP, and ProstT5. Among them, ProtT5 refers specifically to ProtT5-XL-U50 from the ProtTrans series, which adopts a T5-style architecture and contains approximately 3B parameters. The two ESM-2 variants utilize a BERT-style architecture with 3B and 15B parameters, respectively. ESM-3 similarly relies on a bidirectional transformer backbone but differs from ESM-2 in that it is trained as a multimodal generative masked language model over sequence, structure, and function tokens. CARP, in contrast, employs a ByteNet-style architecture and is significantly smaller with only 640 M parameters. ProstT5 differs from the other models in that it incorporates structural information during training. However, this structural signal is encoded in the form of 3Di sequences, allowing the model to remain purely sequence-based. Importantly, obtaining embeddings from ProstT5 still requires only the raw protein sequence as an input. ProstT5 also adopts a T5-style architecture and is obtained by fine-tuning ProtT5-XL-U50, thus also containing approximately 3B parameters.

From Figure 3, we observe that ProtT5 and ProstT5 consistently produce clear and stable diagonal signals, indicating a well-aligned and coherent structural correspondence across the majority of protein pairs. In contrast, ESM-2 (3B) and CARP often generate noisy, grid-like artifacts and fail to capture consistent structural similarity. ESM-2 (15B) demonstrates noticeable improvement over the 3B variant but still exhibits degraded performance in certain cases. For example, the vertical streaks in the 1a05–1j7a protein pair illustrate one such failure mode. ESM-3 (7B) appears to perform at a level broadly intermediate between ESM-2 (3B) and ESM-2 (15B), but its results remain less satisfactory overall. These patterns are consistently observed in the more extensive set of comparisons provided in Figure S1. Taken together, these results suggest that from a qualitative perspective, ProtT5 and ProstT5 are the most robust and reliable models for capturing structural patterns. This may be attributed to the T5-style architecture. Notably, although ESM-2 (3B) shares a similar parameter scale and is also trained with a masked language modeling objective, it performs substantially worse on this task, which suggests that the architecture may be a key contributing factor. This interpretation is further supported by the behavior of ESM-3, which, despite being a multimodal model that performs strongly across a range of tasks, does not appear to yield sequence-derived representations that are particularly effective for this task. This contrast suggests that the T5-style architecture may be one important factor in capturing structure-related signals in this setting.

Further supporting the qualitative findings, the quantitative results in Figure S2 show that ProtT5 and ProstT5 indeed provide a more favorable balance between precision and recall compared with the other models. However, between the two, ProstT5 performs significantly better, achieving noticeably higher precision and F1 score. This indicates that while both models produce heatmaps with clear structural signals, the signals from ProstT5 more accurately correspond to the ground truth aligned regions. Among the pLMs trained purely on sequence data, ProtT5 achieves the strongest performance. In contrast, ProstT5 benefits from the explicit incorporation of structural information during fine-tuning, which enables the model to better learn how to generate embeddings that capture alignment-relevant features. This structural supervision leads to a clear performance gain over ProtT5 on this task. Accordingly, in the subsequent experiments, we use ProstT5 as the pLM for generating residue embeddings.

### Effect of Applying the Sliding Window and the Sigmoid-Based Transformation

To assess the effectiveness and necessity of using the sliding window and sigmoid-based transformation in our pipeline, Figure 4 illustrates how different window sizes and the transformation affect the cosine similarity matrices using the protein pair 1g99–1gqy as an example. The four columns correspond to sliding window sizes of 1, 5, 10, and 15, respectively. The first row shows the raw cosine similarity matrices computed directly from the window embeddings, while the second row presents the transformed matrices, using the sigmoid-based transformation described in Methods. As shown, the strongest signal in the raw matrices, which has a cosine similarity of only around 0.5, is significantly highlighted after transformation in the reward matrices. This enhancement makes the key regions more distinguishable from the surrounding noise. In contrast, noise-prone regions in the top-right and bottom-left corners are strongly suppressed, remaining dark blue to indicate high penalties.

**Figure 4:**
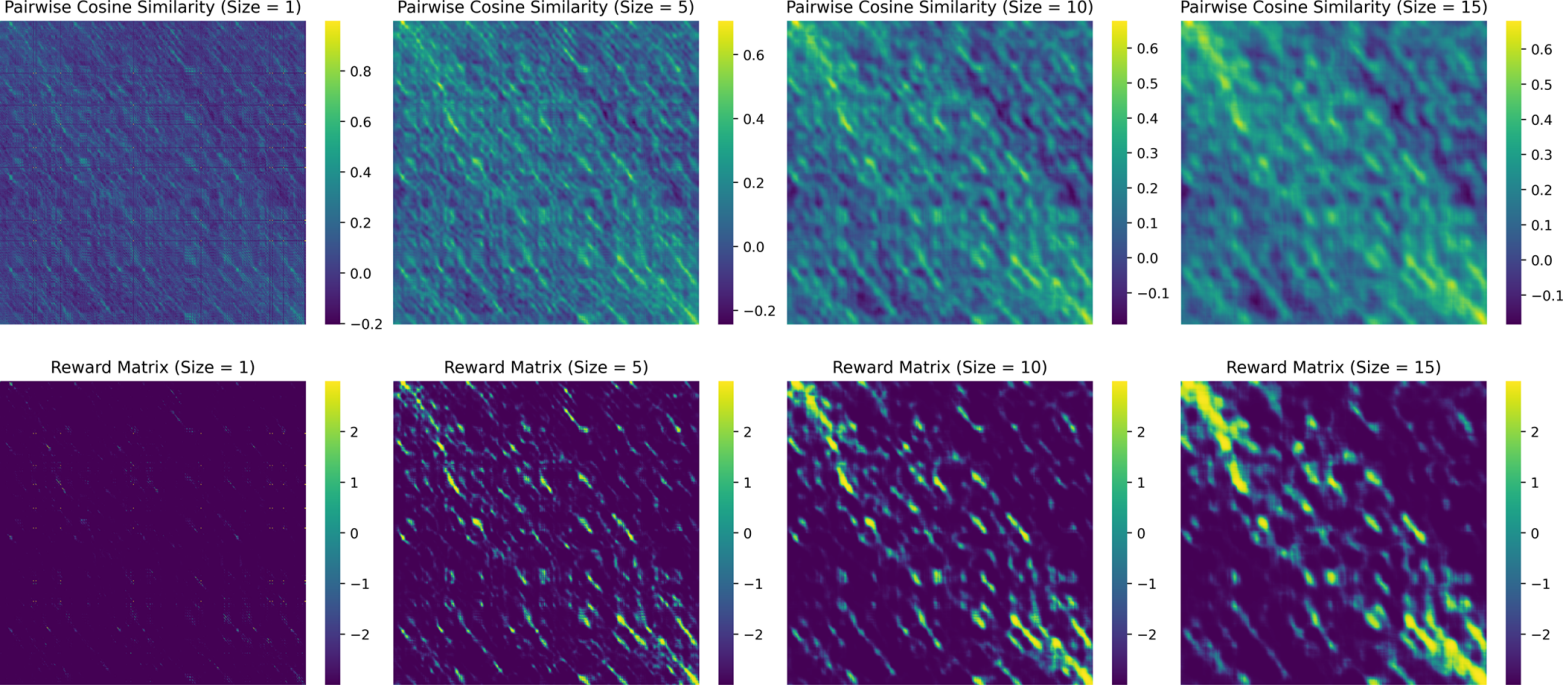
Comparison of cosine similarity matrices before and after sigmoid-based transformation across different sliding windows. The top row corresponds to the pairwise cosine similarity matrices before the transformation for protein pair 1g99-1gqy at different sliding window sizes. The bottom row shows the matrices after the transformation was performed using a sigmoid function.

Without the use of a sliding window, as exemplified in the upper left of the figure (i.e., window size 1), the resulting matrix shows continuous signals that are equal across the grid. Thus, after the transformation, the resulting matrix is left with mostly penalized pairs, leaving little to no continuous signal. Using a sliding window amplifies the signal between sequences within a pair, making the aligned regions stand out from the background noise successfully. A trade-off exists between the size of the continuous signal and the fidelity, where the true signal is preserved more faithfully than that with larger ones, where high cosine-similarity pairs dominate.

In the original raw cosine similarity matrix, many window pairs exhibit nonzero similarity scores, including a substantial amount of noise. The sigmoid-based transformation more precisely isolates truly informative signal regions. This transformation suppresses noisy, low-similarity scores by shifting them below zero, preventing the model from identifying overly large or spurious regions. At the same time, it accentuates high-similarity scores, effectively highlighting salient regions in the reward matrix and enabling more accurate detection by subsequent algorithms.

### Assessment of Sliding Window Identified Similarities Proves Detection of Analogous Structures

To evaluate the strength of the structural signal detected by our method, we first identified candidate aligned region pairs in the MALISAM data set using our proposed workflow, followed by structural alignment to assess their validity. We employed ProstT5 as the pLM to extract embeddings, as our prior benchmarking demonstrated that ProstT5 most effectively encodes fine-grained structural information relevant to our task, outperforming other pLMs in this regard. Regarding hyperparameters, we used a sliding window of size 3. A sigmoid-based transformation was applied with a midpoint of 0.2, a sharpness of 9, and a scaling factor of 3. Details of the hyperparameter selection process are provided in Figure S3. For the alignment step, we adopted an indel penalty of 10, following standard practices in local alignment. For each protein pair in the MALISAM data set, we applied our method to sequences derived from the complete PDB structures and identified the region pair that yielded the highest alignment score under our framework. To assess whether the detected regions are indeed structurally similar, we computed TM-scores for both the full-length protein structures and the corresponding detected regions of each protein pair. These scores reflect global and local structural similarity, and their comparison is shown in Figure 5.

**Figure 5:**
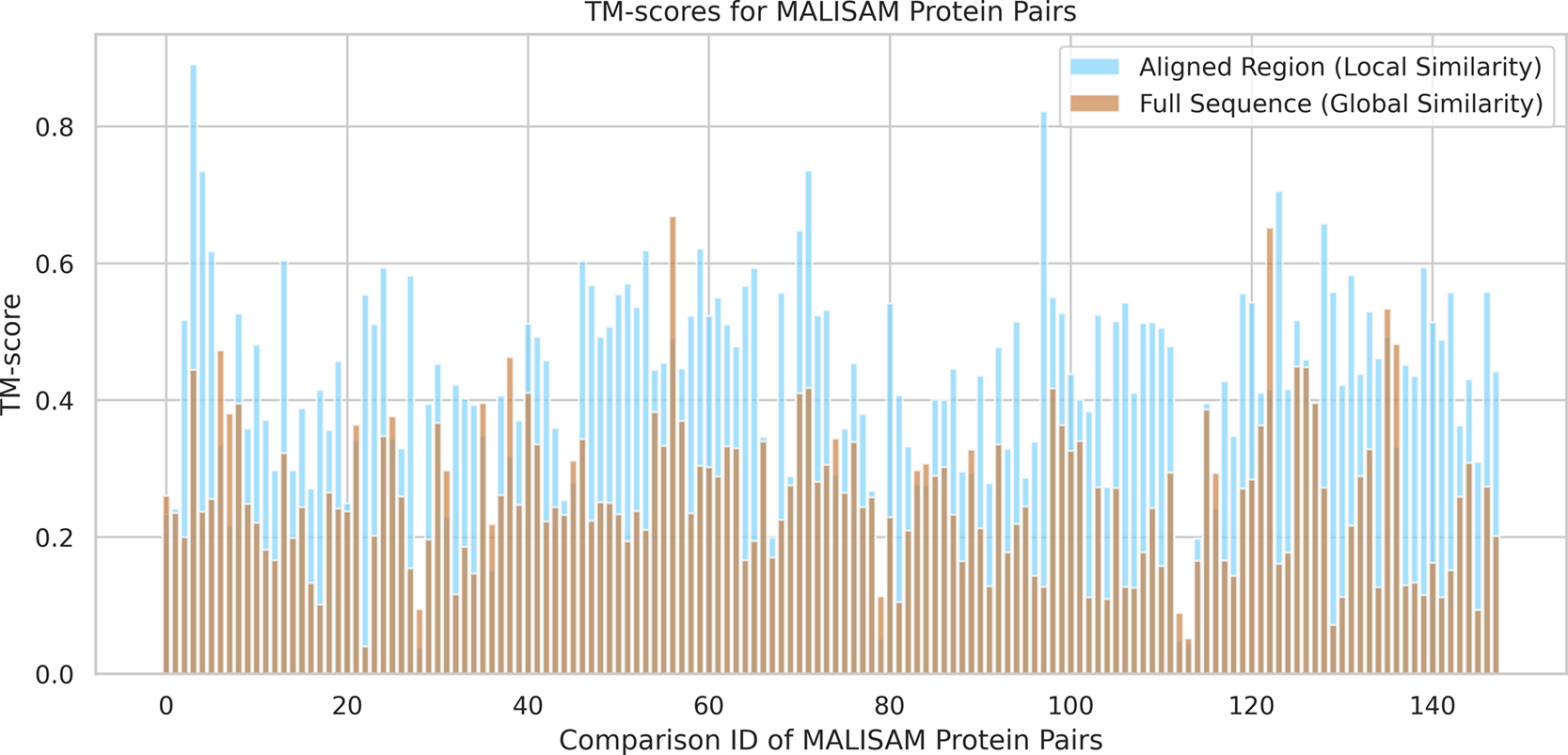
TM-score: full-length structures vs detected regions. Comparison of global and local structural similarity across all protein pairs in the MALISAM data set.

Among the 148 pairs of protein chains in the MALISAM data set that contain aligned local structural regions, the global structural similarity, as measured by the TM-score between the full-length chains, remains low, averaging around 0.25. In contrast, the local similarity scores for the regions detected by our method are significantly higher. Specifically, 93 pairs achieve a TM-score above 0.4, with 53 of them exceeding 0.5. This indicates that our method is indeed capable of detecting aligned regions and motifs that are locally analogous in structure. However, there are also 4 pairs with local similarity scores below 0.1, and tuning the transformation hyperparameters did not lead to improved results for these cases. This suggests that the ability of ProstT5 to capture local structural similarity might be limited in some challenging cases, thereby influencing the downstream performance of our approach.

### Comparison with Residue and 3Di Local Alignment Baseline Methods

To evaluate the effectiveness of our workflow, we compared it against two representative baselines. The first baseline performs standard local alignment directly on raw protein sequences using Biopython’s PairwiseAligner, with the BLOSUM62 substitution matrix and gap penalties set to 11 (gap open) and 1 (gap extension), matching the default settings of blastp. The second baseline involves transforming the amino acid sequences into predicted 3Di sequences using ProstT5, followed by local alignment of the resulting 3Di representations using the same PairwiseAligner. For these 3Di alignments, we used Foldseek’s 3Di substitution matrix (mat3di.out) with gap penalties of 10 (gap open) and 1 (gap extension), matching the default gap costs used in Foldseek’s 3Di-based local alignment.

As shown in Table 1, we compare the average length of the predicted region pairs obtained by each method as well as the number of pairs with TM-scores above or below specific thresholds. When comparing alignments based on amino acid sequences with those based on predicted 3Di sequences, the latter shows significantly better performance. Under comparable predicted length ranges, the method using predicted 3Di sequences produces nearly twice as many region pairs with TM-scores above 0.5 compared to the amino acid-based approach. Moreover, among methods that use the Prost T5 encoder, our sliding window + transformation + Smith–Waterman-like alignment approach outperforms the alternative that first decodes the embeddings into 3Di sequences and then aligns the resulting sequences. This suggests that the continuous embedding space captures local structural information more effectively than the discretized 3Di representation.

**Table 1:** Comparison of Methods Based on TM-Scores and Lengths of Detected Local Regions

	local alignment	ProstT5 predicted 3Di + local alignment	ProstT5 embeddings + our workflow
TM-score < 0.1	4/148	4/148	4/148
TM-score > 0.5	25/148	48/148	53/148
TM-score > 0.4	52/148	83/148	93/148
length	18.3 ± 11.0	19.3 ± 19.3	29.7 ± 16.5

Our method can produce different results depending on the choice of hyperparameters. When the window size is fixed, the three hyperparameters related to the sigmoid-based transformation do not have a single, clearly optimal setting. Instead, they primarily determine where the results fall along the trade-off curve between region length and TM-score. To facilitate a fairer comparison with the other two baselines, we selected a hyperparameter configuration that results in relatively shorter region lengths: a midpoint of 0.2, a sharpness of 9, and a scaling factor of 3. This setting yields a mean region length of around 30 amino acids, which keeps the length reasonably close to those of the other two methods while still effectively capturing biologically meaningful motifs, about 10 amino acids longer than the regions identified by the other baselines. At the same time, our method continues to detect more high TM-score pairs, identifying 10 and 5 additional pairs with TM-scores greater than 0.4 and 0.5, respectively, compared with the method based on predicted 3Di sequences.

Overall, these results validate the effectiveness of our method. They also indicate that information relevant to local structural similarity is already well encoded in ProstT5 embeddings. While decoding the 3Di sequence can indeed make the approach more compatible with the Foldseek toolchain for structural similarity search, it is not necessarily the most effective way to exploit the structural information embedded in the representations.

### Case Study: Specialized Detection of SH3 Domains

To further demonstrate the practical utility of our framework, we performed a case study on SH3 domains, a small but structurally conserved protein domain that plays a central role in mediating protein–protein interactions in signaling and regulatory pathways. SH3 domains are characterized by a compact β-barrel fold composed of five β-strands arranged into two orthogonal β-sheets, forming a surface that recognizes proline-rich peptide motifs.^27,28^ These domains are widely distributed in eukaryotic signaling proteins and often occur in diverse sequence contexts, making them an appropriate system for evaluating whether sequence-derived embeddings can detect locally conserved structural motifs across proteins with substantial sequence variability.

To specialize our detection of SH3 domains, we refined the alignment workflow by adjusting the initialization of the scoring matrix and the logic of the traceback procedure. Specifically, we transitioned from a standard local alignment to a fitting alignment formulation, an algorithmic approach situated between global and local alignment. This strategy constrains the target motif to be aligned from its beginning to its end against a subsegment of a larger candidate sequence. In this case study, we leveraged residues 61–121 of PDB entry 1kik as a canonical target motif. By enforcing this fitting constraint, we ensure that the known structural module is mapped in its entirety to candidate proteins, facilitating the robust discovery of latent SH3 domains across the sequence library.

For this case study, we curated a library consisting of all single-chain proteins with experimentally determined structures from the PDB, which includes 84,360 proteins. Embeddings for the target motif and the PDB subset were generated by using ProstT5. Cosine similarity matrices were computed using a sliding window size of 3, followed by the sigmoid-based transformation with a midpoint of 0.2, a sharpness of 9, and a scaling factor of 3. The alignment stage employed an indel penalty of 10. This framework identifies an aligned range for each protein and records the corresponding raw alignment score. The entire PDB subset was ranked according to these alignment scores. To assess the accuracy of our approach, we evaluated the results against ground truth SH3 domain annotations.

To establish a rigorous ground truth for evaluation, we compiled a comprehensive list of PDB entries containing the SH3 domain by retrieving all experimentally determined structures annotated with InterPro identifier IPR001452 via the SIFTS PDB mapping resource. As illustrated in panel C of Figure 6, we evaluated the precision and recall across the top k-ranked proteins. Our results indicate that the predictions within the top 200 are nearly entirely composed of proteins containing validated SH3 domains. Furthermore, approximately 85% of the proteins containing the SH3 domain in the PDB subset were successfully recovered within the top 500 candidates. While the SH3 domain possesses a relatively simple architecture consisting solely of β-strands, which may simplify the retrieval task, these findings underscore the practical utility of our sequence-based framework for identifying structurally conserved motifs in large-scale protein libraries.

**Figure 6:**
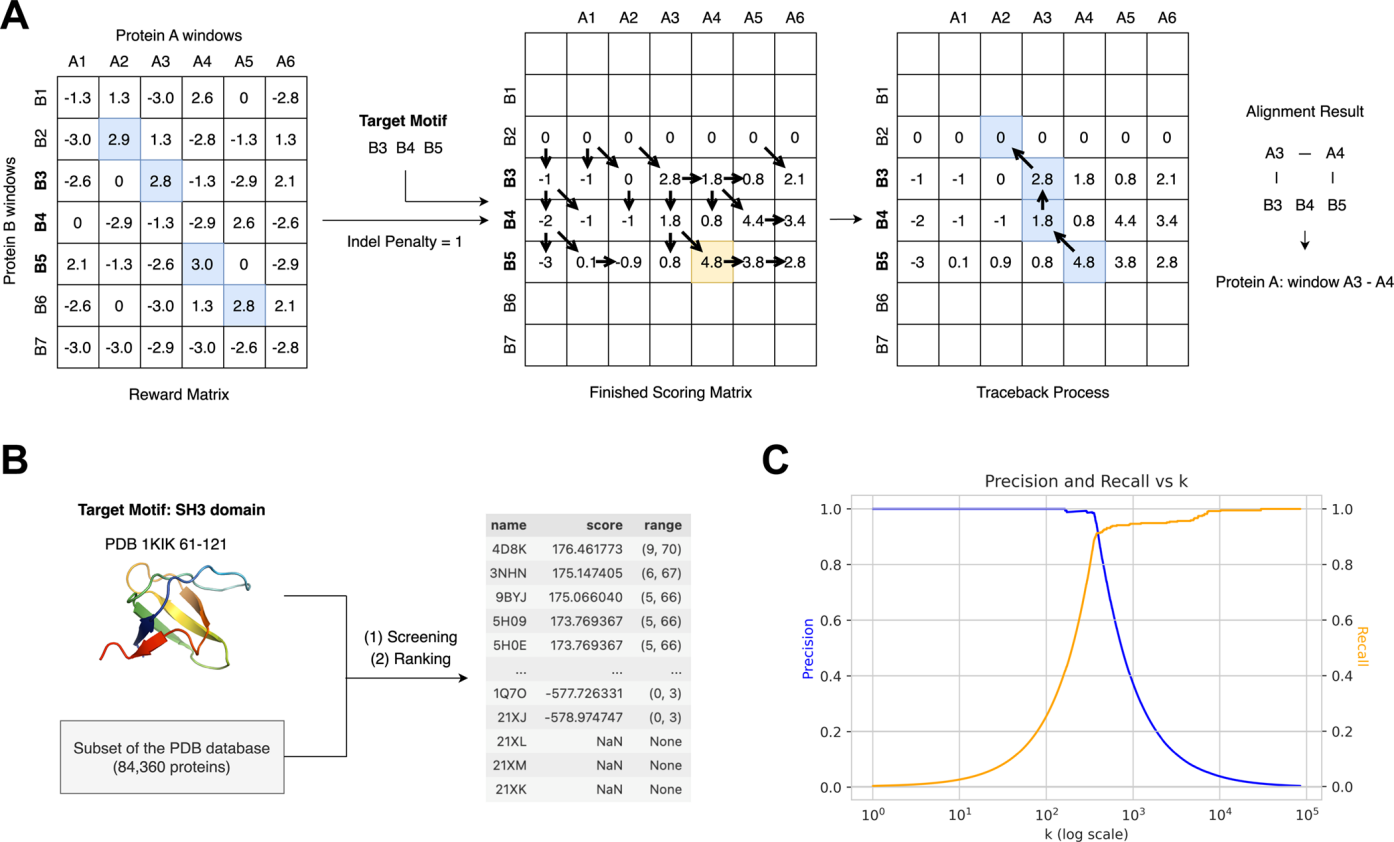
Case study. (A) Alignment using a fitting alignment variant based on a predefined reward matrix. (B) Large-scale screening and ranking using the target motif. (C) Evaluation of ranking results. The pseudocode of the algorithm is provided in Supporting Information, Algorithm 2. It should be noted that parameters displayed in this schematic (e.g., indel penalty = 1) are selected for illustrative clarity and may differ from the values used in the actual experiments (e.g., indel penalty = 10).

## Conclusions

In this work, we investigate the fine-grained structural information encoded in pLM embeddings and explore their potential for detecting local protein structural similarity. We present a scalable and zero-shot framework for detecting structurally aligned regions directly from sequence data and provide initial validation of this approach using a rigorous benchmark, the MALISAM data set, which comprises protein pairs with low sequence similarity. In many cases, the identified regions achieve TM-scores above 0.5, indicating strong structural correspondence.

Our method builds on the key observation that pairwise cosine similarity computed from pLM embeddings using a sliding window can capture structurally analogous regions. As illustrated in the section “Heatmap of Embedding Cosine Similarity Reveals Structurally Analogous Regions”, this signal consistently emerges across protein pairs from different structural classes, suggesting that this methodology is generalizable across protein folds. Among the models evaluated, ProtT5 and ProstT5 yielded the most distinct and reliable alignment patterns, suggesting a strong inductive bias of the T5 architecture toward capturing local structural features.

At the current stage, our framework employs a uniform fixed-sized sliding window. However, recognizing that different window sizes may be better suited for capturing structural information at varying granularities and that distinct secondary structural elements may exhibit unique spatial dependencies, we plan to explore the integration of multiscale or adaptive windowing strategies in future work to further refine the extraction of local structural signals.

When comparing our approach to baseline methods, we find that although both use the same ProstT5 encoder, our framework outperforms the approach that decodes embeddings into 3Di sequences, followed by local alignment. This observation further supports the idea that information relevant to local protein structural similarity is largely preserved in the sequence-derived embeddings themselves. Converting embeddings into 3Di sequences is not the only way to utilize this information and may not be the most effective. Our framework illustrates an alternative approach that makes more direct use of the structural signals encoded in pLM embeddings.

## Supplementary Material



## Data Availability

The source code of this work is available at https://github.com/ZinniaMa/SlidingwinAlignment.

## Notes

Supplementary methods and experiments, including algorithmic details, comparison of patterns across different pLMs, and hyperparameter selection (PDF)
