# Supplementary material for: Inferring Local Protein Structural Similarity from Sequence Alone
Source: J Chem Inf Model. 2026 May 2;66(10):5699–707. doi: 10.1021/acs.jcim.6c00114 (PMC13213830; doi:10.1021/acs.jcim.6c00114)
Supplement: Supplementary file 1 [file ci6c00114_si_001.pdf]

Supporting Information

Inferring Local Protein Structural Similarity from Sequence Alone

Zinnia Ma[1], Javier Espinoza Herrera[2], Elsy Buitrago-Delgado[4], Neville P. Bethel*[2], and Adrian Jinich*[2,3]

[1]Department of Bioengineering, UC San Diego
[2]Department of Chemistry and Biochemistry, UC San Diego
[3]Skaggs School of Pharmacy and Pharmaceutical Sciences, UC San Diego
[4]School of Medicine Division of Regenerative Medicine, UC San Diego

*Emails: nbethel@ucsd.edu; ajinich@ucsd.edu

Contents

Supplementary Methods **S3**
Algorithmic Details S3

Supplementary Experiments **S5**
Supplementary comparison of patterns across different pLMs S5
Quantitative comparison of patterns across different pLMs S7
Hyperparameter selection S8

Supplementary Methods

Algorithmic Details

Algorithm 1: Local Alignment (Smith-Waterman) Using Predefined Reward Matrix

```
                         // Uses predefined reward_matrix_{i,j} instead of symbol comparisons
OutputLCS(backtrack, i, j)
    if i = 0 and j = 0
        return [], []
    if backtrack_{i,j} = "s"
        return [], []
    else if backtrack_{i,j} = "d"
        (o_1, o_2) ← OutputLCS(backtrack, i − 1, j)
        return o_1 + [str(i − 1)], o_2 + ['−']
    else if backtrack_{i,j} = "r"
        (o_1, o_2) ← OutputLCS(backtrack, i, j − 1)
        return o_1 + ['−'], o_2 + [str(j − 1)]
    else if backtrack_{i,j} = "m"
        (o_1, o_2) ← OutputLCS(backtrack, i − 1, j − 1)
        return o_1 + [str(i − 1)], o_2 + [str(j − 1)]

Alignment(reward_matrix, indel_penalty)
    v ← number of rows in reward_matrix
    w ← number of columns in reward_matrix
    s_{0,0} ← 0
    for i ← 1 to v
        s_{i,0} ← 0
        backtrack_{i,0} ← "s"
    for j ← 1 to w
        s_{0,j} ← 0
        backtrack_{0,j} ← "s"
    for i ← 1 to v
        for j ← 1 to w
                          ⎧ s_{i−1,j} − indel_penalty
            s_{i,j} ← max ⎨ s_{i,j−1} − indel_penalty
                          ⎪ s_{i−1,j−1} + reward_matrix_{i−1,j−1}
                          ⎩ 0
            if s_{i,j} = 0
                backtrack_{i,j} ← "s"        // stop: local alignment terminates here
            else if s_{i,j} = s_{i−1,j−1} + reward_matrix_{i−1,j−1}
                backtrack_{i,j} ← "m"        // match: move diagonally from (i − 1, j − 1)
            else if s_{i,j} = s_{i−1,j} − indel_penalty
                backtrack_{i,j} ← "d"        // down: move from (i − 1, j)
            else if s_{i,j} = s_{i,j−1} − indel_penalty
                backtrack_{i,j} ← "r"        // right: move from (i, j − 1)
    bestscore ← max s_{i,j}
    for i ← 1 to v
        for j ← 1 to w
            if s_{i,j} = bestscore
                (o_1, o_2) ← OutputLCS(backtrack, i, j)
                return bestscore, [o_1[0], o_1[−1]], [o_2[0], o_2[−1]]
```

Algorithm 2: Fitting Alignment Using Predefined Reward Matrix

```
                              // Uses predefined reward_matrix_{i,j} instead of symbol comparisons
OutputLCS(backtrack, i, j)
    if i = 0 and j = 0
        return [], []
    if backtrack_{i,j} = "s"
        return [], []
    else if backtrack_{i,j} = "d"
        (o_1, o_2) ← OutputLCS(backtrack, i − 1, j)
        return o_1 + [str(i − 1)], o_2 + ['−']
    else if backtrack_{i,j} = "r"
        (o_1, o_2) ← OutputLCS(backtrack, i, j − 1)
        return o_1 + ['−'], o_2 + [str(j − 1)]
    else if backtrack_{i,j} = "m"
        (o_1, o_2) ← OutputLCS(backtrack, i − 1, j − 1)
        return o_1 + [str(i − 1)], o_2 + [str(j − 1)]

Alignment(reward_matrix, indel_penalty)
    v ← number of rows in reward_matrix
    w ← number of columns in reward_matrix
    s_{0,0} ← 0
    for i ← 1 to v
        s_{i,0} ← s_{i−1,0} − indel_penalty
        backtrack_{i,0} ← "d"
    for j ← 1 to w
        s_{0,j} ← 0
        backtrack_{0,j} ← "s"
    for i ← 1 to v
        for j ← 1 to w
                          ⎧ s_{i−1,j} − indel_penalty
            s_{i,j} ← max ⎨ s_{i,j−1} − indel_penalty
                          ⎩ s_{i−1,j−1} + reward_matrix_{i−1,j−1}
            if s_{i,j} = s_{i−1,j−1} + reward_matrix_{i−1,j−1}
                backtrack_{i,j} ← "m"          // match: move diagonally from (i − 1, j − 1)
            else if s_{i,j} = s_{i−1,j} − indel_penalty
                backtrack_{i,j} ← "d"                        // down: move from (i − 1, j)
            else if s_{i,j} = s_{i,j−1} − indel_penalty
                backtrack_{i,j} ← "r"                       // right: move from (i, j − 1)
    bestscore ← max s_{v,j}
    for j ← 1 to w
        if s_{v,j} = bestscore
            (o_1, o_2) ← OutputLCS(backtrack, v, j)
            return bestscore, [o_1[0], o_1[−1]], [o_2[0], o_2[−1]]
```

Supplementary Experiments

Supplementary comparison of patterns across different pLMs

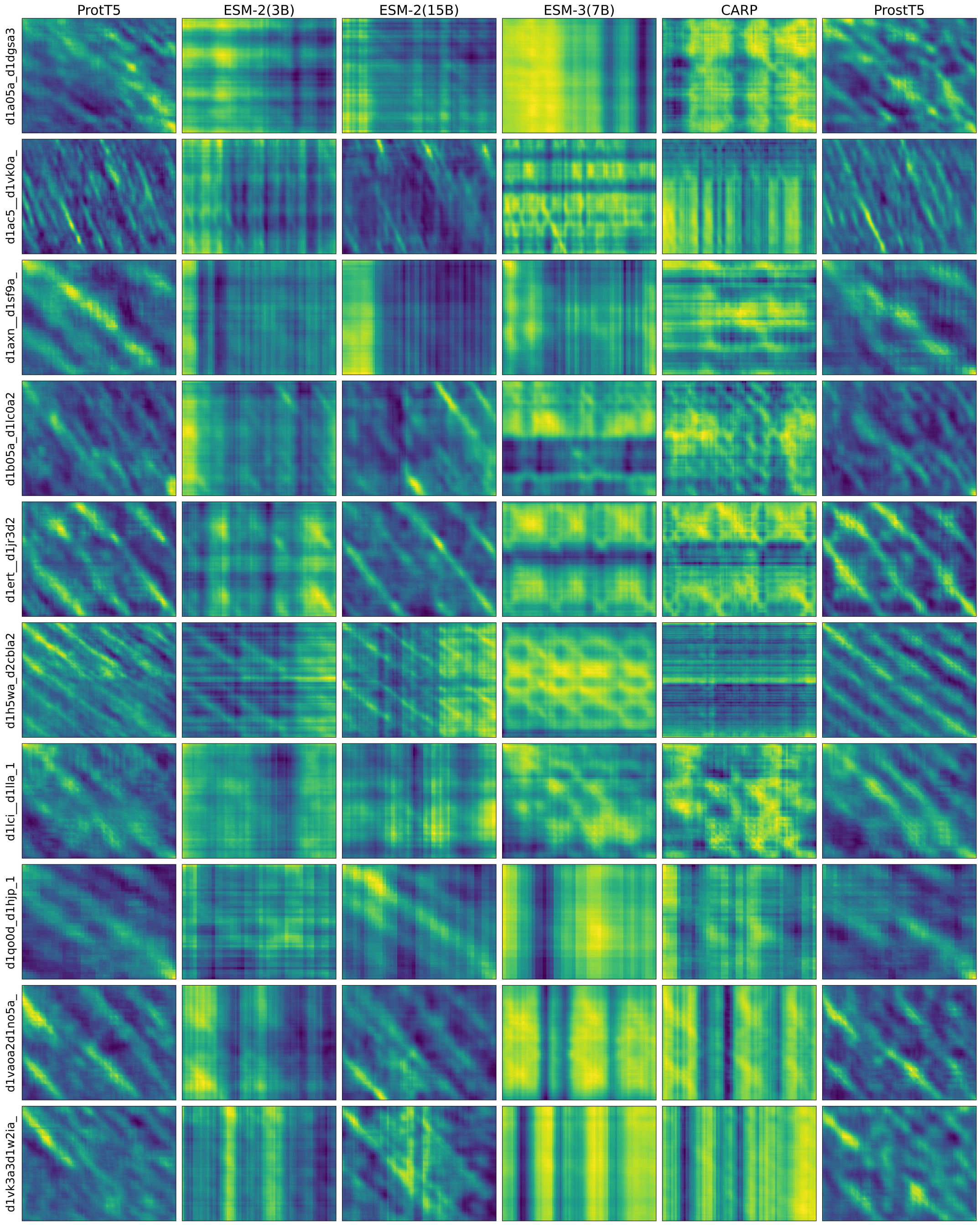

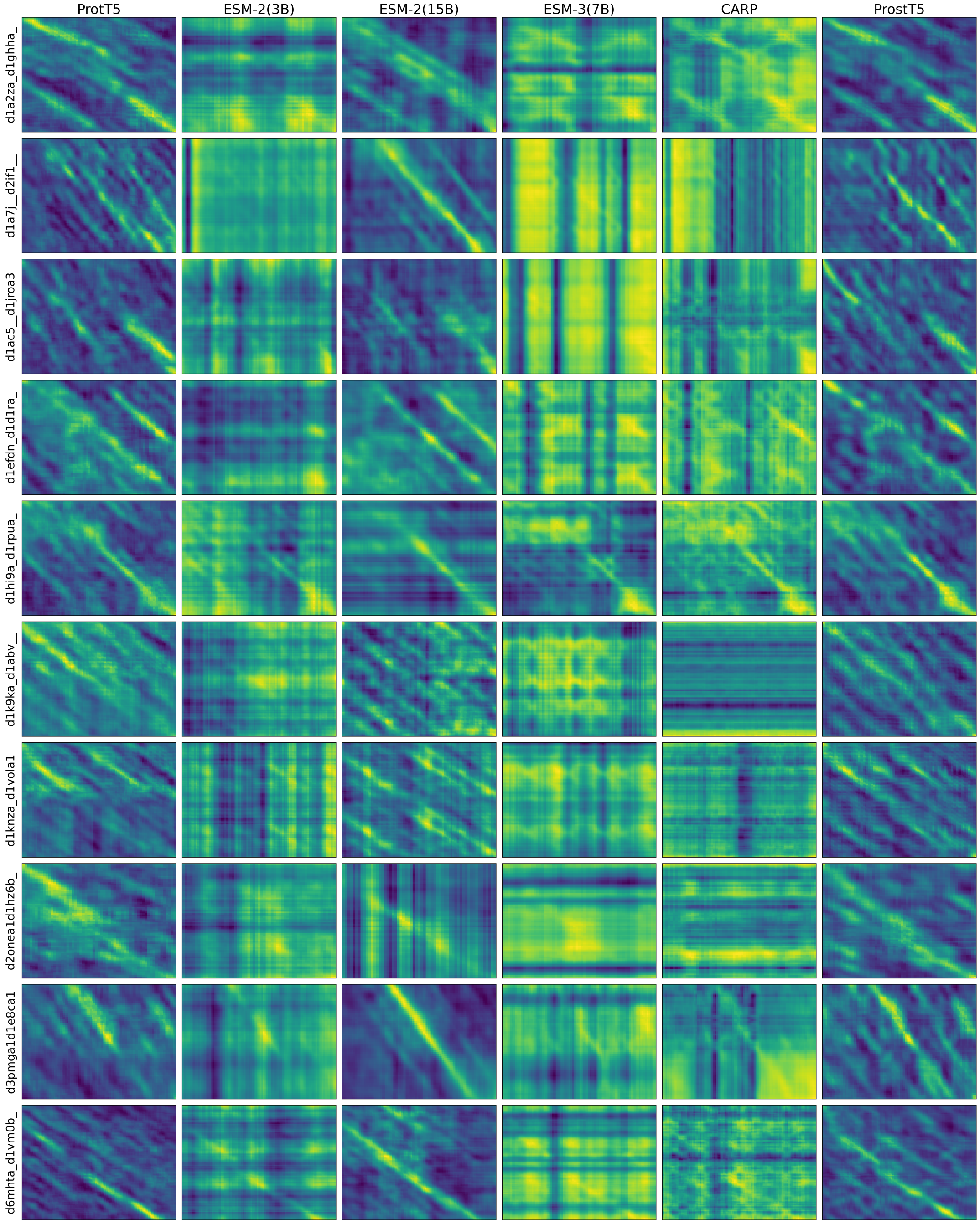

Figure S1: **Extended comparison of pairwise cosine similarity patterns across different pLMs.**

Quantitative comparison of patterns across different pLMs

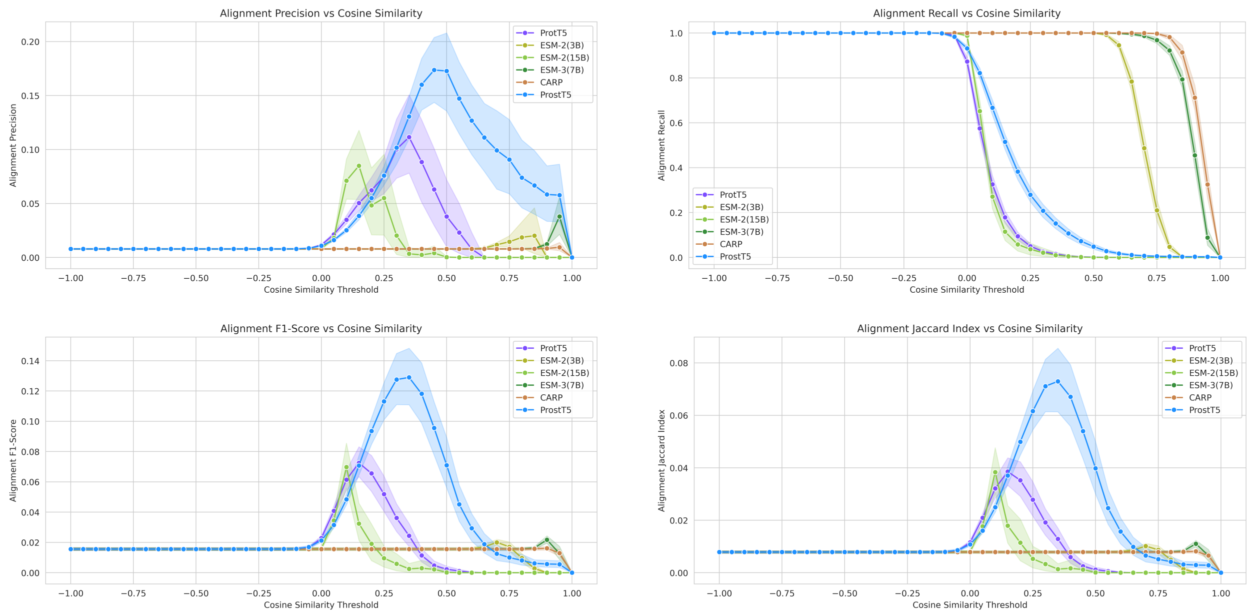

Figure S2: **Pairwise alignment performance across cosine similarity thresholds for different pLMs. Panels show precision (upper left), recall (upper right), F1-score (lower left), and Jaccard index (lower right).**

To quantify how well raw cosine similarity between embeddings can recover known structurally aligned regions, we used the TM-align–based alignments provided in the MALISAM entries as ground truth. For each protein pair, we parsed the corresponding file and generated an alignment matrix in which an entry is 1 only when both residues are aligned and belong to the analogous motif, and 0 otherwise. Independently, for each model, we computed an all-vs-all cosine similarity matrix between per-residue embeddings of the two sequences. Given a cosine similarity threshold, we then predicted residue pairs as aligned wherever the similarity matrix exceeded the threshold, and compared this predicted alignment to the ground-truth matrix to count true positives, false positives, and false negatives at the residue-pair level. From these counts, we computed precision, recall, F1-score, and the Jaccard index for each threshold, model, and protein pair.

Figure S2 summarizes the effect of cosine similarity thresholding on alignment precision, recall, F1-score, and the Jaccard index across the evaluated pLMs. Across all thresholds, the models exhibited the expected trade-off between precision and recall, though their performance profiles differed significantly. At low cosine similarity thresholds, recall remained maximal across models, reflecting the recovery of a large number of non-specific alignments. However, this resulted in very low precision. As the threshold increased, the precision of ProstT5, ProtT5, and ESM-2 (15B) improved modestly, peaking before declining to zero. In contrast, the precision of CARP, ESM-2 (3B), and ESM-3 (7B) barely surpassed the baseline value and declined rapidly. Similarly, the latter two models failed to improve the F1-score and Jaccard index beyond their initial value. The Jaccard index follows the same overall trend as the F1-score across cosine similarity thresholds, reflecting that both metrics are derived from the same counts of true positives, false positives, and false negatives and therefore respond similarly to changes in the predicted alignment set. These results are consistent with the visual patterns observed in the similarity matrices. ProstT5, ProtT5, and ESM-2 (15B) produced alignment-like diagonal regions, whereas CARP, and ESM-2 (3B), and ESM-3 (7B) produced more diffuse straight-line patterns that did not suggest meaningful structural correspondence. Overall, these results suggest that ProstT5 offers the best balance between precision and recall, maintaining signal across a wider range of thresholds than ProtT5 and ESM-2 (15B). In contrast, CARP and ESM-2 (3B) are less effective at capturing local structural similarity from sequences alone.

Hyperparameter selection

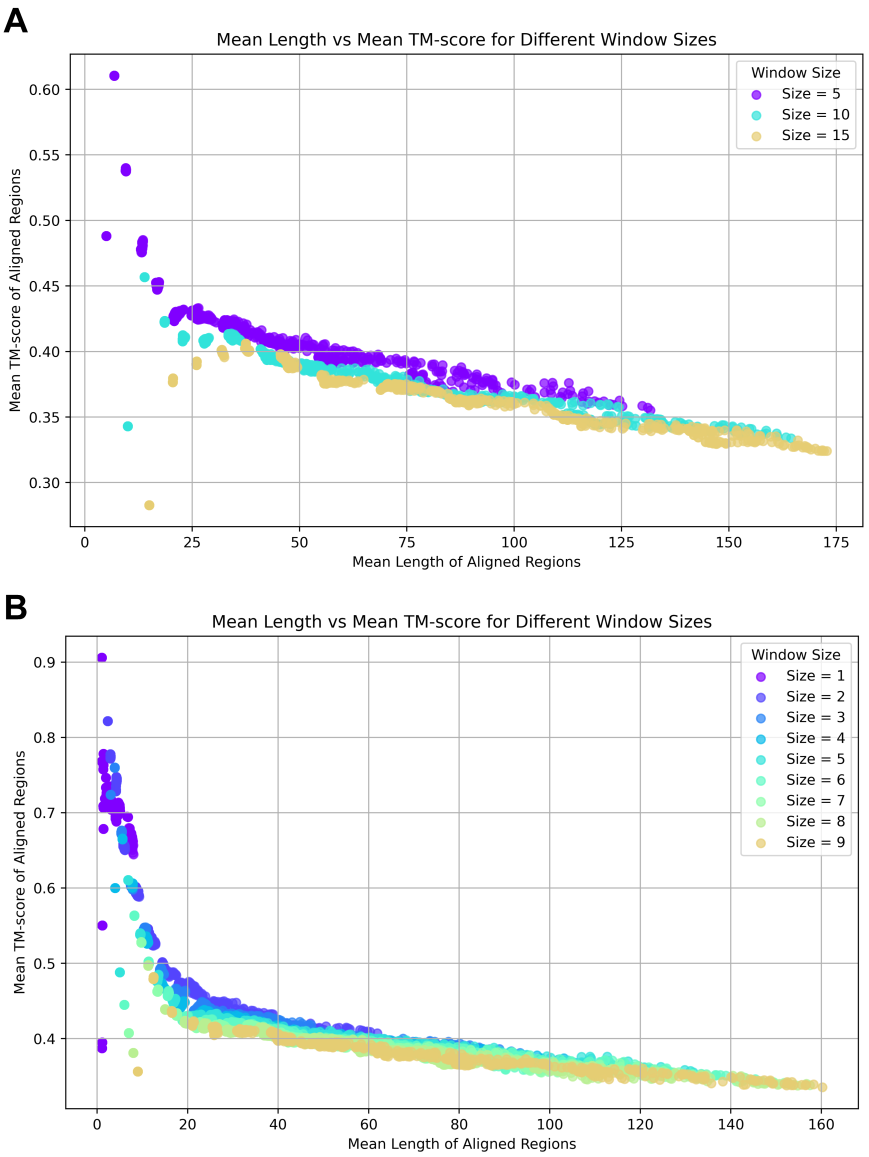

Figure S3: **Grid search results for sigmoid-based transformation hyperparameters across different window sizes.**

To identify the most suitable hyperparameters, we conducted an extensive comparison and search. For each window size, we performed a grid search over combinations of three hyperparameters used in the sigmoid-based transformation: midpoint, sharpness, and scale. Specifically, the midpoint was varied from 0 to 1 with a step size of 0.1, while both sharpness and scale were varied from 1 to 10 with a step size of 1, resulting in a total of 1100 combinations. For each combination, we applied our alignment method to all protein pairs, generating a pair of aligned regions for each, and subsequently computed the region length and TM-score for each pair.

Figure S3 presents results from hyperparameter search experiments. For each hyperparameter combination under each window size, we computed the average region length and TM-score across all protein pairs. Each point in the scatter plot corresponds to one such combination, with the color indicating the window size, as specified in the legend. We observe a consistent trend across the dots corresponding to each window

size: higher TM-scores are generally associated with shorter region lengths. As the hyperparameters are adjusted to make the sigmoid function impose a stricter penalty, this leads to the detection of shorter regions. At the same time, such stricter filtering results in higher TM-scores, reflecting more confident alignments.

When comparing across different window sizes, as shown in Panel A, we find that the distributions of points shift noticeably: for a window size of 5, the points are concentrated more towards the upper-left region of the plot; for a window size of 15, they shift towards the lower-right; and window size of 10 lie in between. This observation is consistent with the results presented in Figure 4. Increasing the window size smooths the signals in the pairwise cosine similarity matrix, reducing the likelihood that small gaps disrupt contiguous aligned regions. Consequently, the detected regions tend to be longer, but this comes at the cost of lower TM-scores. Notably, for a fixed region length, points corresponding to window size 5 consistently achieve higher TM-scores, indicating better alignment quality. Based on this, we consider window size 5 to be a more desirable setting. To further refine our choice, we conducted a finer-grained search around this value, evaluating all window sizes from 1 to 9, as shown in Panel B.

The results reveal that the general trend still holds: smaller window sizes tend to yield points closer to the upper-left region of the plot, reflecting shorter but higher-quality alignments. However, some exceptions are observed. At window size 1, where no sliding window is applied, the lack of signal continuity prevents the detection of region pairs of sufficient length. Consequently, all hyperparameter combinations fail on most protein pairs. With window size 2, some valid regions begin to appear in a subset of cases, but no combination achieves consistent success across all pairs. It is only from window size 3 onward that certain hyperparameter settings yield successful region detections for the full dataset. Notably, starting at window size 5, all tested hyperparameter combinations are able to detect valid regions across all protein pairs.

When the window size is fixed, varying the combination of the three hyperparameters in the sigmoid-based transformation reveals a clear trade-off between detecting regions with higher TM-scores and identifying longer regions. A higher midpoint leads to more areas in the original pairwise cosine similarity matrix being suppressed, effectively classifying them as mismatches and penalizing them accordingly. This results in shorter detected regions. Increasing the sharpness amplifies already prominent signals, which encourages the traceback procedure to favor these stronger regions over other contiguous but weaker segments. A larger scale, on the other hand, amplifies the reward associated with longer regions, making it easier to overcome the fixed indel penalty (set to 10) and thereby reducing the likelihood of interruptions within the aligned region. Overall, there is no universally optimal combination of these hyperparameters, as the best choice depends on the desired balance between alignment accuracy and region length for different applications. In our experiments, we selected the hyperparameter setting that achieved the highest mean TM-score while still detecting valid regions for all protein pairs under a window size of 3. This led us to choose a midpoint of 0.2, a sharpness of 9, and a scaling factor of 3.

We further observed that the number of cases with TM-score greater than 0.5 or 0.4 is positively correlated with the average TM-score, while the number of cases with TM-score below 0.1 consistently remains limited to four specific cases. This suggests that when the pLM captures reasonably accurate underlying information for a given case, hyperparameter tuning can significantly influence the quality of the detected regions. In contrast, when the pLM fails to provide reliable underlying information, downstream tuning becomes largely ineffective. A closer examination of these four cases reveals that three of them involve protein pairs consisting of an artificial protein and a natural protein, suggesting that ProstT5 may still be limited in its ability to represent artificial proteins, which could explain the observed failure cases.

It is noteworthy that the hyperparameter combination selected here is derived from the grid search results on the MALISAM dataset, which carries a potential risk of overfitting to this specific benchmark. When applying this framework to protein datasets with significantly different distributions, such as those with unique structural topologies or conserved motifs of varying lengths, performing initial tuning on a small subset of samples is advisable. Nevertheless, as demonstrated by our case study results, the chosen set of hyperparameters exhibits a foundational level of generalizability.
